# Basement membranes in obstructive pulmonary diseases

**DOI:** 10.1016/j.mbplus.2021.100092

**Published:** 2021-11-12

**Authors:** Bart G.J. Dekkers, Shehab I. Saad, Leah J. van Spelde, Janette K. Burgess

**Affiliations:** a University of Groningen, University Medical Center Groningen, Department of Clinical Pharmacy and Pharmacology, Groningen, The Netherlands; b University of Groningen, University Medical Center Groningen, Groningen Research Institute for Asthma and COPD (GRIAC), Groningen, The Netherlands; c University of Groningen, University Medical Centre Groningen, Department of Pathology & Medical Biology, Experimental Pulmonology and Inflammation Research, Groningen, The Netherlands

**Keywords:** ADAM9, a metalloproteinase domain 9, ASM, airway smooth muscle, BM, basement membrane, Col IV, collagen IV, COPD, chronic obstructive pulmonary disease, ECM, extracellular matrix, LN, laminin, MMP, matrix metalloproteinase, Th2, T helper 2, TIMP, tissue inhibitors of metalloproteinase, VSM, vascular smooth muscle, Asthma, Chronic obstructive pulmonary disease, Laminin, Collagen IV, Airway remodeling, Airway inflammation

## Abstract

Increased and changed deposition of extracellular matrix proteins is a key feature of airway wall remodeling in obstructive pulmonary diseases, including asthma and chronic obstructive pulmonary disease. Studies have highlighted that the deposition of various basement membrane proteins in the lung tissue is altered and that these changes reflect tissue compartment specificity. Inflammatory responses in both diseases may result in the deregulation of production and degradation of these proteins. In addition to their role in tissue development and integrity, emerging evidence indicates that basement membrane proteins also actively modulate cellular processes in obstructive airway diseases, contributing to disease development, progression and maintenance. In this review, we summarize the changes in basement membrane composition in airway remodeling in obstructive airway diseases and explore their potential application as innovative targets for treatment development.

## Highlights

• Basement membrane composition is changed in the airways of patients with obstructive airway diseases.
• Basement membrane changes are linked to disease characteristics in patients.
• Mechanisms behind the altered BM composition remain to be elucidated.
• Laminin and collagen IV affect key pathological processes in obstructive airway diseases.

## Introduction

Chronic respiratory diseases are the third leading cause of death, with approximately 550 million people affected worldwide and 4 million deaths per year [1]. Asthma and chronic obstructive pulmonary disease (COPD) are the most common chronic respiratory diseases.

Asthma is characterized by variable respiratory symptoms, such as wheeze, shortness of breath, chest tightness and cough, and variable expiratory airflow limitation. In addition, it is usually associated with airway inflammation [2]. COPD is a common, preventable and treatable disease that is characterized by persistent respiratory symptoms and airflow limitation that is due to airway and/or alveolar abnormalities caused significant exposure to noxious particles or gases [3].

Both asthma and COPD are associated with cough, dyspnea, mucous hypersecretion, inflammation, airway wall remodeling, and airway hyperresponsiveness. Both inflammation and airway remodeling may lead to airway hyperresponsiveness, defined as an exaggerated obstructive response to various nonspecific stimuli [4].

Airway remodeling, defined by structural changes in the airway architecture, is a characteristic feature of both asthma and COPD. Airway remodeling includes increased airway smooth muscle (ASM) mass, increased vascularity, epithelial shedding, goblet cell hyperplasia and increased and changed deposition of extracellular matrix (ECM) proteins, including basement membrane (BM) proteins [5]. Airway remodeling in asthma occurs in both the large and small airways, whereas remodeling in COPD appears to occur mainly in the small airways (referred to as chronic bronchitis) [6]. Emphysema is an additional feature of remodeling in COPD characterized by abnormal and permanent enlargement of air spaces and destruction of the lung parenchyma distal to the terminal bronchioles [7]. Recent studies have shown that expression and turn-over of BM proteins changes in obstructive airway diseases. Moreover, BM proteins have been shown to play a key role in regulating various aspects of inflammation and remodeling. Current treatment strategies for both asthma and COPD are focused on amelioration of bronchoconstriction and airway inflammation by using bronchodilators and glucocorticosteroids. A subgroup of patients, in particular patients with severe asthma or COPD, are poorly controlled by these drugs. Here, we review the current understanding of the (patho)physiology of BMs in obstructive airway diseases and explore their potential application as novel targets for treatment development.

## Basement membrane proteins in the lung

The BM in the lung is a thin, dense sheet of ECM separating the airway epithelium, endothelial cells or airway and vascular smooth muscle cells from the underlying connective tissue [8], [9]. Initially, electron microscopy showed the BM to consist of three distinct layers, the lamina lucida, the lamina densa, which are collectively known as the basal lamina (the ‘true BM’), and the reticular lamina. Follow-up studies demonstrated that these layers are, however, likely to be an artifact from sample processing and that the BM is actually a single layer [10], [11]. Studies on the expression of specific BM proteins in the airways have thus far focused on the expression of individual laminin chains in the large airways. Studies on the expression of specific laminin heterotrimers, other individual BM proteins in the large airways and expression of (specific) BM proteins in the small airways are lacking to date, making this an area of interest which requires attention.

### Laminins

The lamina lucida is the most superficial layer of the BM underlying the epithelium and the endothelium and is comprised mainly of laminins. Binding of laminins to their receptors on the cell membrane initiates a polymerization process resulting in the formation of the primary network of laminin fibers which is essential for BM formation.

Laminins are a family of cruciform-like glycoproteins composed of five α, three β and three γ chains forming 16 different laminin heterotrimers [12]. Expression of each laminin chain is cell and tissue specific. In the epithelial BM of the airways, laminin chains α2, α3, α5, β1-3 and γ1-2 are primarily expressed, suggesting that all laminin heterotrimers may be assembled, with the exception of α1β1γ1, α1β2γ1, α4β1γ1, α4β2γ1, α2β1γ3 and α4β2γ3. In pulmonary endothelial BMs, laminins α4, α5, β1 and γ1 chains are expressed, whereas laminins α4, α5, β1-2 and γ1 expressed in ASM BMs (Table 1) indicating that laminin α4β1γ1 and α5β1γ1 are assembled by both cell types. In addition, α4β2γ1 and α5β2γ1 appear to be assembled by the ASM cells [12], [13], [14], [15], [16], [17], [18], [19].

**Table 1:** Basement membrane protein expression in obstructive airway disease.

**BM protein**	**Basement membrane location**			Changes in obstructive airway diseases
	**Epithelium**	**ASM**	**Endothelium**	
Laminin				
α1	–	–	–	
α2	+	?	?	Asthma: increased epithelial BM expression
α3	+	?	?	Asthma: increased epithelial BM expression
α4	–	+	+	Asthma: decreased ASM BM expression Asthma: decreased endothelial BM expression
α5	+	+	+	Asthma: increased epithelial BM expression Asthma: decreased ASM BM expression
β1	+	+	+	Asthma: increased epithelial BM expression
β2	+	+	?	Asthma: increased epithelial BM expression COPD: increased ASM and VSM BM expression
β3	+	–	–	
γ1	+	+	+	Asthma: increased epithelial BM expression
γ2	+	?	?	
Collagen IV				
α1	+	+	+	
α2	+	+	+	
α3	+	+	+	Asthma: decreased tumstatin expression
α4	+	+	+	
α5	+	+	+	
α6	+	+	+	

+: expressed, -: not expressed, ?: expression unknown. ASM: airway smooth muscle, BM: basement membrane, VSM: vascular smooth muscle.

### Collagen IV

Collagen IV binds to the primary network of laminins and forms a secondary network, the lamina densa. The collagen IV is the major protein found in basement membranes and forms a network that is assembled from three distinct heterotrimers that comprise the six collagen IV chains (α1-6); the ubiquitously expressed and the most abundant α1α1α2 isoform and the tissue specific α3α4α5 and α5α5α6 isoforms [20], [21]. The different collagen IV isoforms are highly homologous and contain three structurally distinct domains; the amino-terminal (7S) domain rich in cysteine and lysine residues, a major collagenous domain followed by a carboxyl-terminal non-collagenous (NC1) domain, of which the latter is unique for each chain [20], [21]. Treatment of BMs with bacterial collagenases results in the release of a specific fragment with a sedimentation coefficient of 7S, namely the 7S domain [20]. In the lung, expression of all collagen IV chains has been detected in the epithelial, endothelial and smooth muscle BMs [22]. Collagen IV provides the BM with mechanical strength by covalently crosslinking at three sites within its heterotrimeric structure; the N-terminal 7S domain, the C-terminal noncollagenous-1 (NC1) domain and the lateral triple-helical domains [20].

### Other BM components

Nidogens and proteoglycans stabilize the laminin and collagen IV networks [8]. Nidogens cross-link laminins to collagen IV through linkage of the laminin γ1 chain and the triple-helical domains of collagen IV. Nidogens occur in mammals in two isoforms, nidogen 1 and 2. During development, both isoforms are expressed in an overlapping manner. However in adults, nidogen 2 is solely expressed in endothelial BMs, while nidogen 1 is expressed in all BMs [23].

Heperan sulphate proteoglycans further stabilize the BMs [24]. Perlecan, agrin and collagen XVIII form the most prominent members of this group, which have been shown to be expressed in most BMs. Expression of these proteoglycans in the airways has not been specifically examined thus far.

Physiological turnover of BM proteins may result in the formation of matrikines (smaller fragments released from parent ECM molecules that may have functional roles that differ from their parent molecule) [25]. Tumstatin and endostatin, matrikines derived from collagen IV and collagen XVIII respectively, have been shown to be present in the lung and bronchoalveolar lavage fluid of healthy subjects [22], [26]. Expression of other matrikines in the lungs remains to be determined [25], [27], [28], [29].

## Physiological role of BM proteins in the lung

The BM has several key functions, including providing 1) a structural scaffold to support cells, 2) a template for tissue repair, 3) a reservoir for growth factors, 4) a selectively permeable barrier for molecules to cross; forming a physical barrier for cells and proteins; and finally forming an adhesive link between the epithelium/endothelium and the interstitial matrix [24], [30]. In addition to the aforementioned, BM proteins affect cellular function by engaging specific receptors in the cellular membrane, such as the family of integrin receptors [31].

Different BM proteins have specific functions in lung development as well, including directing epithelial branching, smooth muscle differentiation and alveolarization [68]. In adult tissues, BM proteins modulate cell adhesion, migration and differentiation [69].

## BM changes in asthma and COPD.

ECM changes are key in remodeling in both asthma and COPD [32], [33], [34]. Altered expression of collagens, elastin, fibronectin, tenascin, proteoglycans and laminins has been reported in the airways of patients with both asthma and COPD [5], [33], [34]. When thinking about ECM changes in both diseases, the epithelial BM has been a main focus. In both diseases the reticular BM has been shown to be thickened to a similar magnitude, in contrast to healthy airways [35]. Increased fragmentation of the BM has been noted in large airways of patients with COPD, whereas the BM in asthma is compact and more homogeneous compared to control subjects [36], [37]. Changes in specific BM protein expression, however, appears to be different in each disease.

**Fig. 1:**
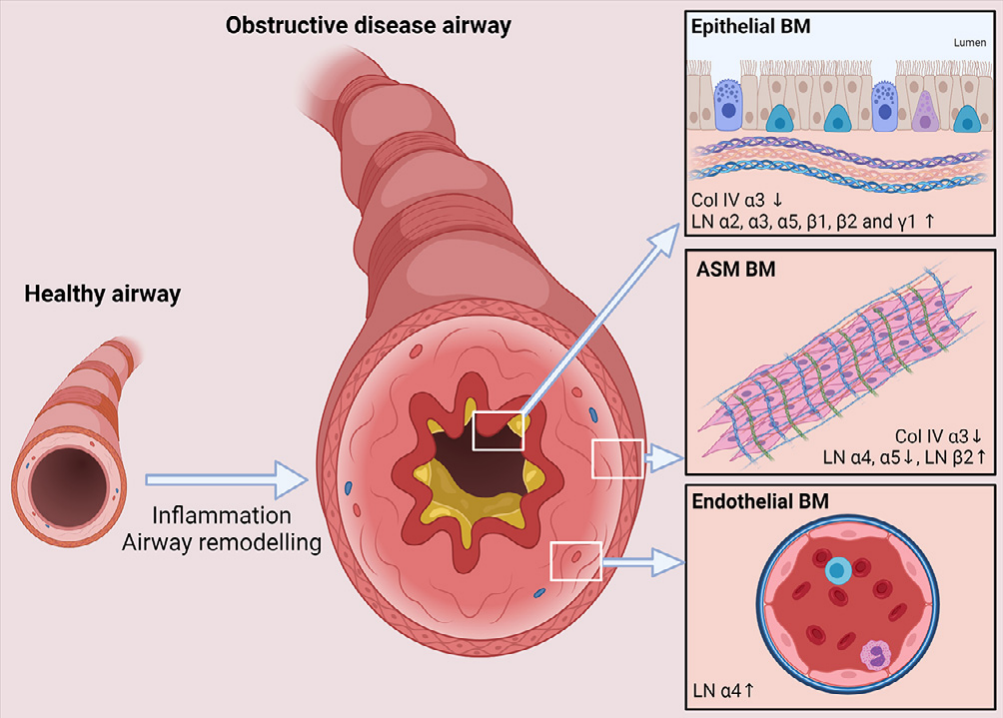
Basement membrane protein expression in obstructive airway disease. Changes in expression of laminins and collagen IV in the epithelial, airway smooth muscle (ASM) and endothelial compartments of patients with obstructive pulmonary diseases. BM: basement membrane, Col IV: collagen IV, LN; laminin.

In the airways of asthmatics, increased expression of several laminin chains, including the α2, α3, α5, β1, β2 and γ1 chain in the epithelial BM has been observed (Fig. 1) [13], [38]. Conversely, expression of collagen IV is decreased in asthmatic patients [39]. More specifically, expression of the collagen IV α3 NC1 domain tumstatin was almost completely absent compared to subjects without asthma, including COPD patients [22]. Expression of endostatin, a C-terminal fragment derived from collagen XVIII, was shown to be increased in the bronchial alveolar lavage fluid of asthmatic subjects, compared to control subjects. Expression of endostatin correlated with expression of vascular endothelial growth factor suggesting a specific role for this protein in vascular remodeling in the asthmatic airways [26]. In the BM of ASM cells of asthmatic patients there was decreased expression of laminin α4 and α5 compared to healthy subjects, whereas laminin α4 expression was increased in the endothelial BMs (Fig. 1)[18]. Reduced expression of laminin α4 in the ASM BM appeared to be related to smoking and no reduction in laminin α4 expression in the ASM was observed between non-smoking asthmatics and control subjects.

In both the large and small airways of patients with COPD, no changes in collagen IV were reported [40], [41]. Similarly, total laminin staining in the epithelial BM of the large airways was comparable between COPD patients and control subjects [35], [41]. Similarly, no changes in laminin expression were observed in lung tissue sections from patients with emphysema [42]. Staining for collagen IV and laminin β2, however, were increased significantly in regions of the large airways where the epithelium was damaged in both healthy subjects and COPD patients (Fig. 1)[41]. Given that, in COPD, epithelial injury is thought to be a chronic process, expression of both BM proteins may be increased. In addition, expression of laminin β2 was increased in the airway and vascular smooth muscle of the large airways of patients with COPD [41]. Increased serum levels of endostatin were shown to be predictive of a lower lung function, exacerbations and systemic inflammation in COPD patients [43]. Heperan sulphate proteoglycan expression has been shown to be decreased in patients with emphysema. Although destruction of tissue occurs in patients with emphysema, relative expression of laminins and collagen IV appears not to be affected, suggesting that destruction of alveolar basement membranes is secondary to destruction of other ECM proteins or cell structures [42], [44].

Changes in BM proteins in patients with obstructive pulmonary diseases have been shown to correlate with disease characteristics. In patients with asthma, increased expression of laminin in the ASM correlated with a reduced reversibility of airway obstruction in response to the β_2_-agonist salbutamol [45]. In line with these findings a higher expression of laminin α4 in the ASM of asthmatics was correlated with an reduced lung function and airway hyperresponsiveness [18]. Also in COPD, higher expression of laminin β2 in the ASM was associated with reduced lung function [41]. Collectively, these observations indicate that increased expression of laminins in, or around, the ASM layer led to a more sensitive, stiffer and less distensible airway. Turnover of laminins may be increased in COPD as well, as serum laminin levels are increased in COPD patients, in particular in patients with an eosinophilic component. Moreover, increased laminin serum levels were also associated with reduced lung function [46]. No correlation was found between serum levels of collagen IV degradation products and emphysema [47].

Although no clear changes in collagen IV deposition were observed in biopsy studies, increased turnover of collagen IV, as indicated by measurement of soluble neo-fragments released from collagen IV, has been shown during exacerbations in COPD [48]. Collagen IV α1 and α3 degradation products, but also collagen IV formation products are increased in serum of COPD patients with stable disease [49], and further increased in exacerbations of COPD compared to stable disease levels [48]. Strikingly, markers for collagen IV turnover in serum were found to correlate with lung function and to be predictive of mortality in COPD [50], [51]. In addition, polymorphisms in the collagen IV α3 gene were correlated with enhanced susceptibility for COPD [52]. Finally, endostatin levels, as a marker of collagen XVIII degradation, are increased in sputum of asthmatic patients and serum of COPD patients [26], [43]. In the latter group, endostatin levels were associated with systemic inflammation, exacerbations and lung function.

## Mechanisms responsible for abnormal BM composition

Maintenance of the ECM composition in the lung is a tightly controlled process. Production, secretion and incorporation of proteins in the ECM is balanced by degradation by proteases, such as matrix metalloproteinases (MMPs), cysteine proteases and serine proteases. An additional layer of regulation is provided by the endogenous inhibitors of these enzymes; the tissue inhibitors of metalloproteinases (TIMPs), serpins and cystatins. In the airways, turnover of some ECM proteins is estimated to occur at a rate of more than 10% per day [53].

Laminins are produced and secreted by a variety of structural and inflammatory cells [54]. In mature tissues, expression of laminin mRNA, especially that of the laminin α chains, is low, suggesting that laminin turnover occurs at a very slow rate under normal circumstances. Turnover of laminins has even been suggested to be linked to the turnover of the cells producing them [55]. Inflammatory stimuli, however, have been shown to increase expression of laminin α4 by endothelial cells. Expression was increased in response to pro-inflammatory mediators, such as lipopolysaccharide, interleukin-1β and tumor necrosis factor-α, whereas expression was decreased by the angiostatic steroid hydroxymethylprogesterone [56], [57]. Similarly, expression of laminin α5 may be increased by interleukin-1β and tumor necrosis factor-α, while its expression was also increased by hydroxymethylprogesterone [56]. In line with these observations, increased laminin α4 expression by the ASM is associated with the presence of eosinophils in asthmatic patients [18].

Laminins are degraded by serine proteases and MMPs [58], [59]. In particular, expression of A disintegrin and a metalloproteinase domain 9 (ADAM9), which can be produced by monocytes, macrophages and neutrophils and is increased in asthma and COPD patients, may contribute to reduced laminin expression in these diseases [58], [60], [61]. Various proteases have been recognized to contribute to collagen IV degradation [62], [63]. MMP-9 cleaves the α3 chain of collagen IV [64]. Expression of MMP-9, however, is increased in both asthma and COPD, whereas the collagen IV α3 chain is only decreased in asthma [22], [65], suggesting other proteases may also contribute to the degradation of collagen IV. MMP-12 is another potential candidate to be involved in collagen IV turnover as increased levels of a MMP-12-derived collagen IV degradation product have been reported in serum of COPD patients [48], [50], [51], [66]. Various cathepsins are expressed in the lung and increased in obstructive lung diseases, however, these proteases were thought to be unlikely to be involved in the dysregulation of tumstatin expression in asthma [63].

Turnover may not only result in changed expression patterns of BM proteins in the tissue, but may also expose matricryptic sites, biologically active sites within the ECM molecules protein sequence that are normally not exposed in the mature deposited ECM. In addition, degradation of ECM proteins results in the formation of matrikines. Both matricryptic sites and matrikines may activate (patho)physiological processes which are independent from the parent molecule and contribute to inflammation and remodeling. For review of matrikines in lung health and disease the reader is referred to [25], [27], [28], [29].

Collectively, these observations indicate that expression and degradation of BM proteins is tightly balanced by various mechanisms, but maybe be deregulated by external factors, such as inflammation. The precise mechanisms underlying the observed BM changes in asthma and COPD remain to be elucidated.

## Role of BM proteins in obstructive airway diseases

**Fig. 2:**
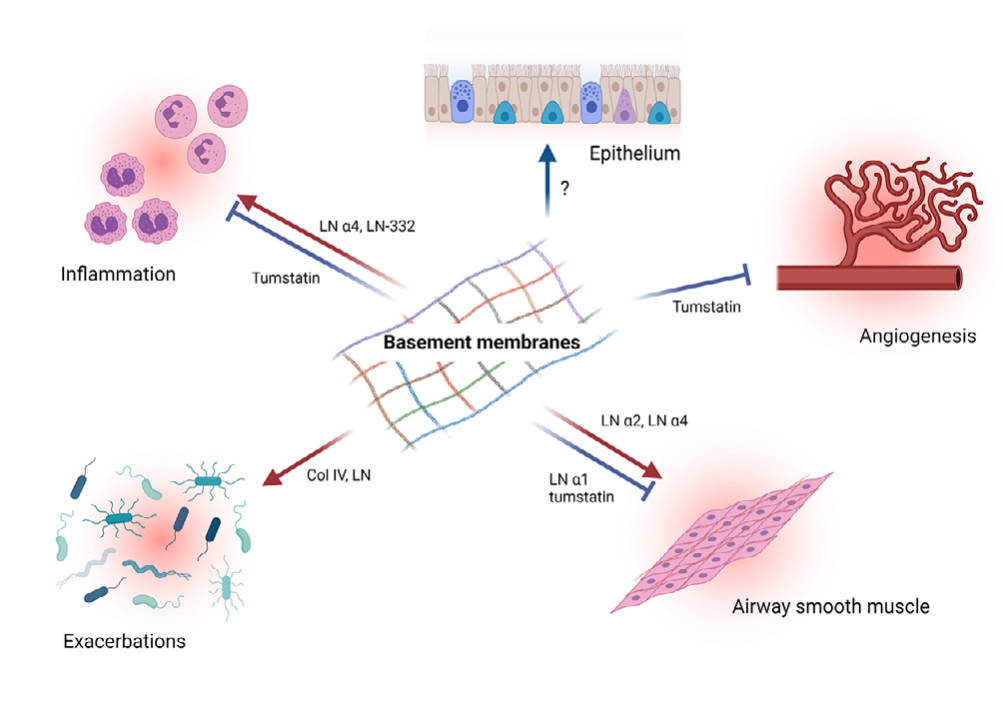
Basement membrane regulation of obstructive airway disease. Laminin α4 promotes airway inflammation and airway smooth muscle (ASM) abnormalities in vitro and in vivo. Similarly, laminin α2 support ASM abnormalities in vitro and in vivo as well. Although only in vitro, laminin α1 has been shown to inhibit ASM phenotype switching. Conversely, the collagen IV derived matrikine tumstatin has been shown to inhibit inflammation, abnormal ASM extracellular matrix production and angiogenesis. Col IV: collagen IV, LN; laminin.

In addition to their physiological role in the lung, BM proteins have also been shown to drive pathophysiological processes in asthma and COPD (Fig. 2). The role of the BM in these processes will be described in the following sections.

### Airway inflammation

Airway inflammation is key in both asthma and COPD pathogenesis and is considered to contribute to variable airway hyperresponsiveness, induction of airway remodeling and to the development, progression and maintenance of disease. Inflammation in asthma is heterogeneous. Thus far, focus has been mainly on type 2 inflammation involving eosinophils, mast cells, T helper 2 (Th2) lymphocytes and elevated immunoglobulin E levels. The mechanisms involved in non-type 2 inflammation are less well characterized [67]. Inflammation in COPD is dominated by neutrophils, macrophages, B-cells, lymphoid aggregates and CD8 + T lymphocytes. The scale of the inflammatory response increases with disease severity [68].

Most evidence about the role of laminins in inflammation comes from studies in the vasculature. Neutrophils, basophils, eosinophils and mast cells have been found to be able to interact with laminins [54]. These interactions result in the binding of these cells to the BM and consequential support or inhibition of pro-inflammatory processes. For a detailed review on the effect of laminins on inflammatory cell function please refer to [54].

*In vivo*, extravasation of leukocytes occurs mainly at sites with high laminin α4 expression and little or no laminin α5 expression [30], [69], [70]. Transmigration across the endothelial BM is presumed to be the rate-limiting step in leukocyte extravasation [71]. Knock-out of laminin α4 results in an ubiquitous laminin α5 expression and is associated with accumulation of T-lymphocytes on the luminal side of the BM [30]. In other inflammatory models, neutrophil and monocyte infiltration is decreased in laminin α4 deficient mice [69]. Both acute and chronic allergen-induced eosinophilic inflammation, induced in a mouse model of asthma were reduced when the animals were treated with a laminin α4 function blocking antibody indicating that laminin α4 also promotes inflammatory responses in obstructive airway diseases (Fig. 2)[18]. In line with these inhibitory effects of laminins on airway inflammation, treatment with the laminin-competing peptide Tyr-Ile-Gly-Ser-Arg (YIGSR) enhanced eosinophilia in a guinea pig model of allergic asthma [72]. In these studies, however, no effect of laminin α5 function blocking antibodies on airway inflammation was found.

Increased turn-over of collagen IV may also affect inflammatory responses. Degradation of ECM proteins may result in the formation of matrikines. Tumstatin, a matrikine derived from collagen IV α3, inhibits eosinophilic inflammation in mouse and sheep models of asthma associated with reduced airway hyperresponsiveness, potentially via reduction in blood vessel area and vascular endothelial growth factor expression [22], [73]. In addition, tumstatin and derived fragments may also inhibit neutrophil migration (Fig. 2)[74], [75]. Similarly, treatment with recombinant endostatin, a matrikine derived from collagen XVIII, has been shown to prevent allergen-induced airway hyperresponsiveness, airway inflammation and expression of inflammatory mediators [76]. Matrikines derived from laminin-332 have been shown to promote regenerative alveologenesis [77]. In addition, laminin-332 derived matrikines have been shown to be chemotactic for neutrophils. The relevance of these matrikines for obstructive lung disease is currently unknown [78].

### Airway remodeling

#### Epithelium

Epithelial changes including epithelial denudation, epithelial desquamation and goblet cell hyperplasia have been shown to be characteristic features of airway remodeling in asthma and COPD [79], [80]. These changes also result in a loss of epithelial integrity. Epithelial BMs have been recognized to play an essential role in epithelial repair processes [81]. Despite this observation, studies on the effects of BM proteins on epithelial cell function are limited (Fig. 2). Collagen IV has been shown to support airway epithelial cell attachment, proliferation and differentiation, whereas laminin has been shown to promote migration of epithelial cells [82], [83]. Increased BM thickness beneath the epithelium has been suggested to act as an additional barrier against penetration of foreign particles. Increased BM thickness, however, has also been suggested to promote, rather than prevent allergen sensitization suggesting that changes in epithelial BM composition could drive disease progression [84]. In line with this suggestion, thickening of the epithelial BM is already observed in young children with recurrent lower respiratory symptoms and correlated with use of inhaled corticosteroids [85].

#### Exacerbations

Epithelial denudation and fragmentation of the BM leaves exposed BM and ECM proteins, with potentially neo-epitopes that are usually hidden within the BM/ECM structural network. The exposure of structural BM proteins may increase the chance of bacterial infections caused by micro-organisms, such as *Haemophilus influenzae* and *Moraxella catarrhalis*, and *Aspergillus* species [86], [87], [88], [89]. These species can actively bind to laminin and collagen IV and changes in the BM may support colonization (Fig. 2). Altered composition of the ECM of patients with COPD compared to healthy subjects has been suggested to impact the lung microbiome affecting both inflammatory responses and infections resulting in exacerbations and disease development and progression [90]. Other studies however, also indicated protective effects of ECM proteins in host defense suggesting the role of the ECM in regulating responses to exposure to viral or bacterial pathogens is a complex one [91].

#### Airway smooth muscle

Increased ASM mass is a characteristic feature of airway remodeling in asthma and COPD [5]. In addition, contractile forces generated by ASM may be increased in patients with asthma compared to ASM from non-asthmatic subjects. Both factors may contribute to airway hyperresponsiveness in asthma and COPD [92]. These diverse functions may be the result of ASM phenotype plasticity enabling these cells to switch between contractile, synthetic and proliferative states. ASM remodeling is induced by multiple factors, including ECM proteins, growth factors, cytokines and chemokines [93]. ASM cells obtained from asthmatic patients produce an altered ECM profile compared to cells obtained from healthy subjects, which increase ASM proliferation and cytokine production [94], [95]. This altered ECM profile, includes a reduced expression of laminin α1 and increased expression of laminin α2 in culture [94], [96]. *In vitro* studies on the effects of specific laminin isoforms on ASM cells have shown that laminin α1 chain inhibits ASM phenotype switching and maintains a contractile phenotype, whereas laminin α2 chains promotes a hypercontractile phenotype [72], [97], [98], [99], [100]. Moreover, laminin α2 also inhibits ASM apoptosis providing an alternative way for laminins to increase ASM mass in vivo [96]. Laminin α4 is the most abundantly expressed laminin in vitro and contributes to induction of a pro-contractile, pro-fibrotic and pro-proliferative ASM phenotype. Expression of laminin α5 is much less pronounced. The role for this laminin is more subtle and involves the regulation of the laminin α4 and contractile proteins mRNA expression [18]. *In vivo*, knock out of laminin α2 protected against allergen-induced airway hyperresponsiveness, which was associated with reduced ASM accumulation and normalization of ASM apoptosis [96]. Similarly, administration of antibodies blocking the interaction between laminin α4 and α5 and their respective integrin receptors prevented allergen-induced ASM accumulation [18]. Treatment with the laminin-competing peptide YIGSR, a peptide shown to inhibit the effects of laminin on ASM cells in vitro, also inhibited ASM accumulation in vivo [72], [96], [99], [101]. In addition, this peptide induced a hypercontractile phenotype indicating that both laminin α2 and α4 containing isoforms may contribute to ASM abnormalities in asthma (Fig. 2).

Collagen IV deposition by asthmatic ASM cells is reduced compared to non-asthmatic cells [102]. Expression of collagen IV is increased by asthmatic ASM cell in response to rhinovirus particles, whereas this was not the case for non-asthmatic ASM cells [103]. No effects were observed for β_2_-agonists on collagen IV expression or for collagen IV on β_2_-agonist signaling [104], [105]. Studies on the effects of collagen IV on ASM cell function are mainly limited to the effects of collagen IV matrikine tumstatin. Expression of collagen IV α3 chain by both asthmatic and non-asthmatic ASM cells was found to be absent. Exposure of asthmatic ASM cell to tumstatin resulted in the deposition of an ECM that suppresses endothelial cell and neutrophil chemotaxis [74], [75]. In asthmatic ASM specifically, exposure to tumstatin induced an ECM that inhibited migratory capabilities associated with the changed expression of specific genes, suggesting that the lack of tumstatin expression in the airways of asthmatics may contribute to an ASM-derived ECM which promotes angiogenesis and inflammation [74].

#### Angiogenesis

Changes in the airway wall in asthma and COPD are associated with vascular changes and angiogenesis [106]. Inflammatory and structural cells have been shown to secrete various angiogenic factors, which in combination with local hypoxia, result in the induction of angiogenesis. Angiogenesis may result in edema, subendothelial BM thickening, increased permeability and increased trafficking of inflammatory cells. Various growth factors and cytokines may be released from both structural and inflammatory cells to promote angiogenesis [106].

Both ECM proteins and ECM fragments have been shown to play a crucial role in regulating angiogenesis under physiological and pathophysiological conditions [107]. In the airways, tumstatin has been shown to be co-localized with vascular endothelial growth factor, a principal pro-angiogenic mediator. In these studies, tumstatin was also shown to prevent angiogenesis and expression of vascular endothelial growth factor in a mouse model of asthma (Fig. 2)[22]. Also in a sheep model of asthma, tumstatin prevent allergen-induced angiogenesis and vascular endothelial growth factor immunostaining [73]. *In vitro*, treatment of ASM cells with tumstatin inhibited the formation of a pro-angiogenic ECM [74]. Although expression of endostatin has not been described in obstructive airway diseases, treatment with recombinant endostatin prevented airway hyperresponsiveness, airway inflammation and expression of inflammatory mediators in a mouse model of allergic asthma [76]. The role of laminins in angiogenesis in the airways remains to be determined.

## Future directions

Despite its apparent thin presence providing a junction between two zones in many tissue structures the BM still holds many secrets that may be key for understanding the pathogenesis of obstructive lung diseases. Through looking to other fields or disease focuses we can see there are exciting avenues of investigation to pursue for obstructive airway diseases.

Recent studies have illustrated that the BM is a highly dynamic environment, with a relatively stable scaffold of collagen IV and laminins supporting a highly motile array of players including nidogen, members of the fibulin family, agrin, spondin, and peroxidasin [108]. These smaller matrikines and ECM associated molecules can be recruited from biological fluids and rapidly perfuse the BM scaffold. The infiltration of these matrikines have the potential to change the cellular responses to the BM. In asthma the presence of fibulin-1 in the ECM deposited by ASM cells promotes cell proliferation, particularly in ASM from asthmatic donors. When the ASM cells are prevented from incorporating fibulin-1 in the ECM this pro-proliferative effect is lost. Interestingly, the absence of fibulin-1 did not alter the ASM cells migratory capacity [109]. Whether the spatiotemporal arrangement of the BM is critically different in asthma and/or COPD in comparison to healthy airways remains to be explored.

Epithelial cell interactions with the BM are important for establishing cellular polarization [110], which facilitates the establishment of an impermeable barrier between the lumen and in interior of the airways. Changes in the stiffness of the BM, which is impacted by the ECM composition and protein assembly and crosslinking, can impact the polarization state of the epithelial cells. The biomechanical environment of the BM will also impact many other cellular responses, including growth, differentiation, migration and inflammatory state. Again, these are all elements that we have describe above as being influenced by the BM constituents in obstructive lung diseases. The influence of the ECM and the biomechanical environment that it generates has been considered to a greater extent when thinking about the mesenchymal cells that reside in the interstitial matrix [111], than the direct influence of the BM. However, the cross-talk between the mesenchymal compartment and the epithelial cells is well recognized to contribute to the disease pathology in many lung diseases. The proximity of the BM within this cross-talk zone makes it a prime candidate to dictate outcomes in these conversations, and it logically follows that alterations in the BM will have vast implications for the balance in this system. While little is known about these effects in asthma or COPD it is highly likely that this will be an important element in the ongoing disease process in the lungs.

## Concluding remarks

Garnering further insight about the role of the BM niche that influences all the cells that come into contact with it (including cells that reside within or alongside it and those that traffic through it) in obstructive lung disease pathogenesis will open new possibilities for developing novel therapeutic strategies.

## Funding

JKB was supported by a Rosalind Franklin Fellowship co-funded by the University of Groningen and the European Union.

### CRediT authorship contribution statement

**Bart G.J. Dekkers:** Conceptualization, Investigation, Writing – original draft, Visualization, Supervision. **Shehab I. Saad:** Conceptualization, Investigation, Writing – original draft. **Leah J. van Spelde:** Conceptualization, Investigation, Writing – original draft. **Janette K. Burgess:** Conceptualization, Writing – review & editing, Supervision.
